# The Role of Lipid Alteration in Multiple Sclerosis

**DOI:** 10.3390/ijms27020812

**Published:** 2026-01-14

**Authors:** Agnieszka Damiza-Detmer, Małgorzata Pawełczyk, Andrzej Głąbiński

**Affiliations:** Department of Neurology and Stroke, Medical University of Lodz, Ul. Zeromskiego 113, 90-549 Lodz, Polandandrzej.glabinski@umed.lodz.pl (A.G.)

**Keywords:** lipid alteration, dyslipidemia, multiple sclerosis, LDL, HDL, cholesterol

## Abstract

Multiple sclerosis (MS) is traditionally recognized as a chronic immune-mediated disorder of the central nervous system (CNS), but increasing evidence suggests that systemic metabolic alterations may also contribute to its pathophysiology. Lipid abnormalities in MS have recently attracted renewed research interest, with studies focusing both on dysregulation of lipid signaling pathways and on alterations in standard lipid profile components, including total cholesterol (TC), low-density lipoprotein (LDL), high-density lipoprotein (HDL), triglycerides (TG), and non-HDL cholesterol. Although disturbances in serum lipid profiles are consistently reported in patients with MS, their origin remains unresolved. Emerging data indicate that dyslipidemia may stem from aberrant cholesterol metabolism within the CNS, secondary to demyelination and myelin sheath destruction, leading to the release of lipid-rich debris and subsequent systemic metabolic imbalance. These lipid changes appear to correlate with blood–brain barrier (BBB) dysfunction, suggesting a link between peripheral lipid metabolism and CNS inflammation. This review summarizes current knowledge on the mechanisms underlying dyslipidemia in MS, its potential impact on disease progression, and its relevance as a possible therapeutic or biomarker target in future translational studies.

## 1. Introduction

Multiple sclerosis (MS) is a chronic immune-mediated disorder with autoimmune and neurodegenerative components affecting the central nervous system (CNS) [1]. Current evidence indicates that MS pathogenesis arises from the interplay between immune activation, endothelial dysfunction, and metabolic disturbances, rather than isolated immune mechanisms [1,2,3,4]. The mechanisms described above lead to the destruction of neuronal myelin sheaths within the CNS, a process referred to as demyelination. This process underlies the pathophysiology of MS. MS comprises four clinical subtypes. Relapsing-remitting MS (RRMS) is the most common (≈80%) and features relapses with recovery, though over 40% have incomplete remission and accumulating disability. About 50–60% of RRMS cases transition to secondary progressive MS (SPMS). Primary progressive MS (PPMS) affects 10–15% of patients and involves a steady neurological decline without relapses. Progressive-relapsing MS (PRMS) combines progressive worsening with intermittent relapses [1,2]. Effective monitoring of disease progression remains difficult and necessitates integrative strategies that incorporate clinical assessment, advanced neuroimaging, and molecular biomarkers [5].

Autoreactive immune cells constitute a fundamental component of MS immunopathology, with CD4+ and CD8+ T lymphocytes—particularly Th1 and Th17 subsets—playing a pivotal role in mediating injury t endothelial cells comprising the blood–brain barrier (BBB) [6]. Activated B lymphocytes likewise traverse the disrupted BBB and differentiate into plasma cells that generate autoantibodies targeting not only myelin oligodendrocyte glycoprotein (MOG), myelin basic protein (MBP), myelin-associated glycoprotein (MAG), and proteolipid protein (PLP), but also lipid membrane components, including cholesterol [6,7,8]. BBB disruption is a key feature of MS and correlates with disease severity, although it remains uncertain whether endothelial dysfunction originates within the CNS or peripherally, and whether it is a primary response to oxidative stress or secondary to immune activation [2,9,10,11].

Recent studies increasingly highlight peripheral lipid alterations in MS, although results remain inconsistent. Due to impermeability of BBB, the CNS synthesizes cholesterol locally and transports it via apolipoprotein E (apoE) in high-density lipoprotein (HDL)-like particles through ATP-binding cassette transporter A1 (ABCA1) [12,13]. Excess cholesterol is converted to 24S-hydroxycholesterol (24S-OHC), which crosses the BBB and activates liver X receptors (LXRs), regulating cholesterol handling in both CNS and periphery [14,15]. Demyelination disturbs this system and is reflected in altered circulating lipid parameters such as total cholesterol (TC), HDL, low-density lipoprotein (LDL), and triglycerides (TG) [3,16,17,18]

Particle-size abnormalities and links between small HDL particles and disability in progressive MS have been reported [16], while cholesterol pathway biomarkers correlate with cognitive and motor performance [19]. Genetic analyses suggest RAC2-mediated cholesterol-independent pathways may reduce MS risk, whereas lifelong elevated HDL-C may increase susceptibility [20]. Other studies demonstrate associations between HDL-C and brain atrophy [15], TC and disability in RRMS [21], and dysfunctional HDL in RRMS, particularly in low-BMI individuals [22].

Collectively, these observations underscore the unresolved question of whether dyslipidemia in MS represents a downstream consequence of CNS demyelination, an active contributor to disease pathophysiology, or a potentially modifiable therapeutic target. This review integrates current evidence on lipid disturbances in MS to elucidate mechanistic interactions between central and systemic lipid homeostasis and to assess their emerging diagnostic and therapeutic implications.

## 2. Cholesterol Metabolism in CNS

Cholesterol is the most abundant lipid in the CNS, derived from intestinal absorption and de novo synthesis. Systemically, synthesis occurs in the liver, CNS, adrenal glands, and reproductive organs. Because cholesterol does not cross the BBB, CNS metabolism is largely independent of peripheral pathways. Cholesterol is produced mainly by oligodendrocytes and astrocytes, with minimal neuronal contribution. Oligodendrocytes play a central role in CNS cholesterol metabolism, as they are responsible for the synthesis and incorporation of large amounts of cholesterol required for myelin formation and maintenance [23,24]. Myelin damage and oligodendrocyte dysfunction in multiple sclerosis lead to the release of cholesterol-rich membrane fragments, profoundly altering local lipid homeostasis within the CNS [25]. Experimental studies demonstrate that demyelination is accompanied by dynamic changes in cholesterol handling, including enhanced esterification and storage of excess cholesterol, whereas efficient remyelination requires tightly regulated cholesterol recycling and redistribution [23,25]. Synthesis peaks during postnatal myelination, when oligodendrocytes supply cholesterol for myelin formation, while in the mature CNS astrocytes become the primary source, delivering lipoprotein-associated cholesterol to neurons [12,13,26].

Cholesterol transport within the CNS relies on lipoproteins whose composition differs from serum due to BBB impermeability. CSF lipoproteins contain apoE, apoA1, and apoJ but lack apoB, and structurally resemble enlarged HDL-like particles [27,28]. Their assembly depends on ABC transporters, particularly ABCA1 and ABCG1, which mediate ATP-dependent cholesterol efflux and generate nascent apoE-containing particles [12,13]. AapoA-I is synthesized predominantly in the liver and intestine; however, increasing evidence indicates that apoA-I is not merely a passive peripheral protein but can actively traverse the BBB and participate in central nervous system lipid homeostasis [29]. Owing to its amphipathic helical structure, apoA-I efficiently binds and transports lipids across the BBB, supporting cholesterol efflux, lipid redistribution, and the formation of lipid-poor HDL-like particles within the brain parenchyma and cerebrospinal fluid (CSF) [29,30,31]. Through these mechanisms, apoA-I contributes to the maintenance of neuronal membrane integrity and facilitates the clearance of potentially neurotoxic lipids [29]. In addition, apoA-I interacts with CNS-specific enzymes and receptors, thereby modulating neuroinflammatory and oxidative stress pathways [30]. Importantly, increased apoA-I levels detected in serum and CSF of patients may also reflect enhanced BBB permeability and increased CNS cholesterol turnover associated with demyelination and neuroinflammatory activity, rather than de novo CNS synthesis of apoA-I [29,30,32]. Notably, apoA-I may exert context-dependent immunomodulatory effects, displaying both pro- and anti-inflammatory properties, which together support CNS homeostasis rather than simply reflecting BBB disruption [29]. These mature through esterification, involving enzymes such as cholesterol ester transfer protein (CETP) and lecithin-cholesterol acyltransferase (LCAT), and are taken up by neurons, microglia, and meningeal cells via low-density lipoprotein receptor (LDLR) family members such as LDL receptor-related protein 1 (LRP1), underscoring the central regulatory role of apoE [13,33].

Cholesterol homeostasis also requires elimination of excess intracellular cholesterol. Emerging evidence indicates that the balance between free cholesterol and cholesterol esters is a critical determinant of inflammatory responses and cellular lipid toxicity [34]. While direct clinical data on the free cholesterol–to–cholesteryl ester ratio in multiple sclerosis remain limited, experimental studies of demyelination suggest that excess myelin-derived cholesterol can promote cholesterol ester accumulation within glial cells, particularly microglia, as part of a regulated lipid-handling response [25]. These observations support the concept that altered cholesterol esterification may represent a downstream consequence of demyelination and neuroinflammation in MS, even though this mechanism has not yet been validated as a clinical biomarker. Cholesterol cannot be enzymatically degraded; instead, it is esterified or converted into oxysterols by sterol hydroxylases, most of which belong to the cytochrome P450 family. The principal pathway for cholesterol elimination from the CNS is conversion by CYP46A1 into 24S-OHC. Due to its increased solubility, 24S-OHC crosses the BBB and enters the circulation [12]. Reduced concentrations of 24S-OHC have been reported in neurodegenerative disorders such as Alzheimer’s disease, whereas elevated levels occur during acute MS relapses, reflecting active myelin injury [15,35]. Experimental modulation of CYP46A1 expression demonstrates that its inhibition leads to accumulation of neurotoxic metabolites, whereas increased expression confers neuroprotective effects [15].

Beyond their transport function, oxysterols serve as signaling molecules regulating lipid metabolism. They act as endogenous ligands for LXRs which activate transcription of genes promoting cholesterol efflux, including ABCA1 and ABCG1, thereby reducing intracellular cholesterol accumulation. LXRs also upregulate genes involved in fatty acid synthesis, and additionally influence immune responses. LXR activation in T cells reduces CNS infiltration, limits Th17 differentiation, suppresses pro-inflammatory cytokines, and promotes anti-inflammatory mediators such as IL-10 and TGF-β, demonstrating the convergence of lipid metabolism with immune regulation [4,14,15].

**Figure 1:**
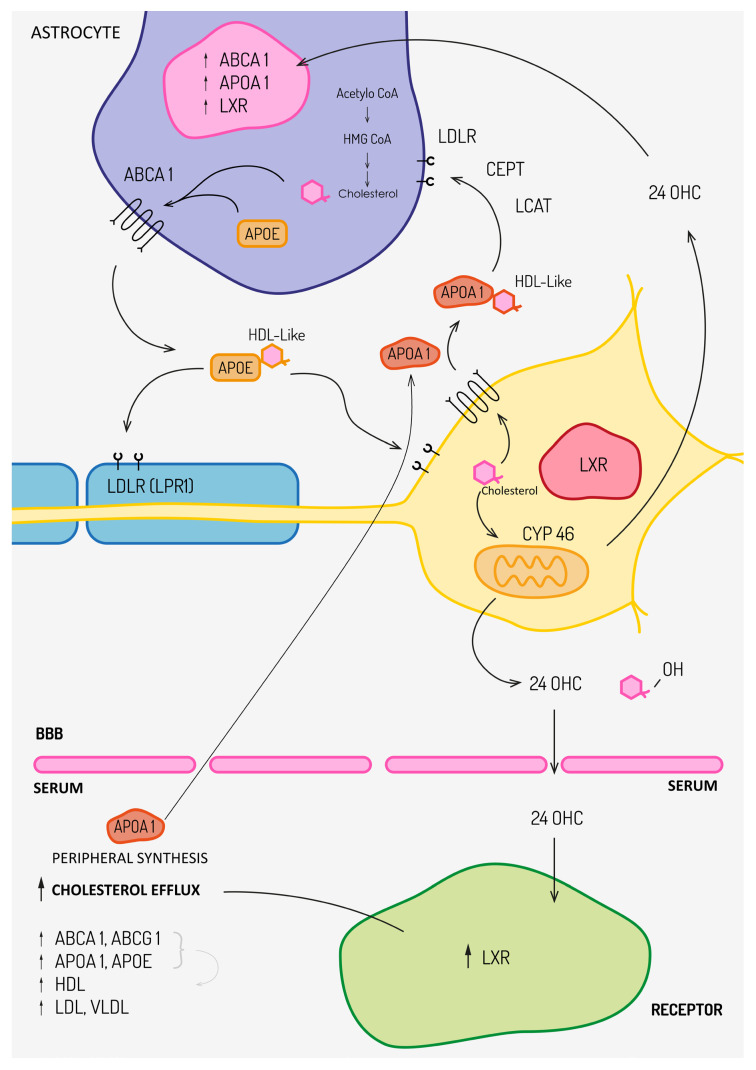
Astrocytes synthesize brain cholesterol, which is exported via ABCA1 as apoE-containing HDL-like particles that bind LDLRs such as LRP1. ApoA-I is synthesized predominantly in the periphery and can traverse the BBB to modulate CNS lipid homeostasis; it is not produced locally within the CNS. Cholesterol is hydrolyzed or converted by CYP46A1 to 24S-OHC, which crosses the BBB and activates LXRs, upregulating ABCA1/G1 and apoE, and increasing peripheral HDL, LDL, and VLDL. Abbreviations: 24S-OHC—24S-hydroxycholesterol; ABCA1/G1—ATP-binding cassette transporter A1/G1; apoE, apoA—apolipoprotein E/A; BBB—blood–brain barrier; CYP46A1—cholesterol 24-hydroxylase; LDLR—low-density lipoprotein receptor; LRP1—LDLR-related protein 1; LXRs—liver X receptors.

Figure 1 summarizes cholesterol metabolism in CNS.

## 3. Lipid Metabolism in MS

A growing body of evidence shows that MS is accompanied by disturbances in lipid metabolism, although it remains unclear whether these abnormalities initiate CNS demyelination or reflect downstream consequences of ongoing autoimmunity. Considering that lipids are major constituents of myelin and essential signaling molecules, clarifying their involvement in MS pathogenesis is of substantial importance.

Understanding the mechanisms underlying dyslipidaemia in MS requires appreciation of CNS lipid compartmentalization. The brain-specific oxysterol 24S-OHC, produced by CYP46A1 activity, crosses the BBB and serves as both a marker of CNS cholesterol turnover and a ligand for LXRs α/β. LXR activation promotes cholesterol efflux by upregulating ABCA1 and ABCG1 expression and increases apoE production, actions that facilitate reverse cholesterol transport. In serum, 24S-OHC is mainly carried in LDL (≈70–80%) and to a lesser extent in HDL (≈20–30%), and is ultimately cleared in the liver via bile acids [36,37]. Under physiological conditions, these pathways maintain cholesterol homeostasis. However, ongoing myelin breakdown in MS can overload CNS cholesterol handling, driving increased CYP46A1 activity and elevated 24S-OHC generation; several studies have documented higher 24S-OHC concentrations in MS and positive correlations with disease duration and MRI lesion burden [38,39]. Thus, part of the serum TC and LDL elevation observed in MS may derive from direct release of cholesterol from damaged myelin and subsequent systemic redistribution.

LXR activation occurs both centrally and peripherally (notably in the liver); oxysterol ligands such as 24S-OHC induce transcription of genes encoding ABCA1, ABCG1 and apoE, promoting reverse cholesterol transport and, paradoxically, contributing to increases in peripheral LDL and VLDL. Upregulation of apoE reflects an adaptive response to facilitate cellular cholesterol efflux. LXRs also stimulate SREBP-1c transcription, promoting fatty acid synthesis [40]. Beyond lipid metabolism, LXRs exert immunomodulatory effects: LXR activation on T cells reduces their CNS infiltration, inhibits naïve CD4+ differentiation into Th17 cells, suppresses IL-9-producing CD8+ populations, downregulates proinflammatory gene expression (TNF-α, IL-1β, IL-6, inducible nitric oxide synthase) and promotes anti-inflammatory mediators such as IL-10 and TGF-β [40,41]. These combined anti-inflammatory and pro-remyelinating actions position LXRs as attractive therapeutic targets in MS.

The humoral response, increasingly recognized as central to MS mechanisms, has driven efforts to identify antibodies directed against myelin and lipid components. While myelin-targeting antibodies such as those against MOG, MBP, MAG, and PLP remain primary candidates [42,43,44], several studies have identified anti-lipid antibodies in patients with MS, including antiphospholipid [45] anti-GalC and anti-GalCer [46], antiganglioside [47], anti-sulfatide [48], and anti-sphingomyelin antibodies [49]. However, none appears specific to MS, and many overlap with other autoimmune and neurological diseases, limiting their diagnostic relevance.

Disruption of sphingolipid metabolism also contributes significantly to MS pathology. Sphingolipids influence neuroinflammation and neuronal survival and are implicated in various neurodegenerative disorders [50]. They modulate immune responses, including apoptosis of autoreactive T cells [49]. Among them, sphingosine-1-phosphate (S1P) is particularly well characterized, exerting pro-inflammatory and neurotoxic effects [51,52]. Increased expression of S1P receptors—especially S1PR1, S1PR2, S1PR3, and S1PR5—has been demonstrated in astrocytes and oligodendrocytes of patients with MS [4,52]. The efficacy of ozanimod, a selective S1PR1/S1PR5 modulator used in RRMS, further underscores the mechanistic importance of sphingolipid signaling and its therapeutic target [53].

Beyond structural lipids, increasing evidence indicates that lipid-derived signaling mediators are altered in multiple sclerosis and may contribute to disease modulation. In particular, components of the endocannabinoid system, including 2-arachidonoylglycerol and palmitoylethanolamide, have been shown to be dysregulated in peripheral immune cells of MS patients. Notably, treatment with dimethyl fumarate was associated with dynamic changes in endocannabinoid levels over time, suggesting that licensed immunomodulatory therapies can influence lipid signaling pathways linked to inflammation and immune regulation. These findings support the concept that lipid signaling, rather than cholesterol levels alone, represents a biologically relevant dimension of lipid dysregulation in MS [54].

MS is associated with alterations in additional lipid classes, including phospholipids, oxysterols, cholesterol esters, and lipid-derived signaling mediators, as summarized in Table 1.

## 4. Peripheral Lipid Alterations in MS and Their Clinical Correlates

Multiple clinical and translational studies describe alterations in peripheral lipid profiles among people with MS. The pattern is heterogeneous, but converging evidence links dysregulated cholesterol metabolism with MRI activity, neurodegeneration and clinical measures of disability.

### 4.1. Cholesterol, LDL and Clinical Measures of Severity and Progression

Multiple independent cohorts demonstrate that elevated TC and LDL concentrations correlate with greater clinical severity in MS, reflected in higher Expanded Disability Status Scale (EDSS) scores, longer disease duration, and faster progression [3,17,18,20,68].

These associations suggest that adverse lipid profiles mirror both inflammatory activity and cumulative neurodegeneration. Elevated LDL and TC may partly reflect the extent of demyelination and axonal injury, potentially amplified by the pro-inflammatory actions of oxidized LDL (ox-LDL) once the BBB is compromised.

Interpretations of these lipid abnormalities generally fall into two mechanistic categories. One view proposes that dyslipidaemia represents a primary peripheral contributor to CNS inflammation. However, epidemiological evidence does not support this: two large cohorts totaling nearly 200,000 women found no association between habitual dietary fat intake and MS risk [69] and interventional studies using hypolipidaemic nutritional agents such as epigallocatechin gallate or coconut oil did not reduce TC or LDL levels in MS [70]. Mendelian randomization (MR) analyses likewise show no causal influence of genetically elevated LDL-C or cholesterol-lowering mechanisms mimicking statins on MS susceptibility or severity [20,71].

It is important to emphasize that classical lipoproteins measured in clinical practice, such as LDL cholesterol, apoB, and the apoB/apoA-I ratio, originate from the peripheral circulation and reflect systemic lipid metabolism. In contrast, the dominant lipoprotein particles within the CNS are HDL-like particles containing apolipoprotein E, which are locally produced and functionally distinct from peripheral lipoproteins [29,72,73,74]. Accordingly, associations between serum lipid parameters and clinical or radiological outcomes in MS should be interpreted as indirect markers of systemic lipid status and disease-related processes, rather than as direct measures of intrinsic CNS lipoprotein biology.

A plausible alternative hypothesis is that increased TC and LDL are downstream consequences of chronic neuroinflammation and perturbed CNS cholesterol metabolism due to myelin injury. In support of this, several imaging and biomarker studies have reported correlations between TC/LDL levels and the burden of demyelinating lesions detected by MRI. Associations have been described between higher LDL and increased numbers of contrast-enhancing lesions (CELs), greater overall lesion counts, and more new T2 lesions [3,15,75,76]. Similar relationships have been reported for elevated TC and brain-specific cholesterol metabolites—24S-OHC and 27-OHC—with lesion activity. Moreover, higher TC has been linked to reduced brain volume in MS cohorts [39,56].

Additional evidence that lipid alterations are closely tied to MS pathobiology comes from observations that disease-modifying and immunomodulatory therapies alter lipid profiles. For example, interferon-β-1a initiation has been associated with early reductions in TC, LDL, TG and HDL during the first weeks of therapy [61], implying that systemic lipid patterns are sensitive to the immune milieu and its pharmacological modulation.

Elevated LDL may also play a pathological role once the BBB is disrupted. LDL particles entering perivascular CNS compartments can undergo oxidative modification, generating ox-LDL, which drives foam cell formation, monocyte recruitment, and release of proinflammatory cytokines such as TNF-α, IL-6, and IL-8 [77,78]. Observational data support positive relationships between LDL/ox-LDL and clinical markers including EDSS, disease duration and lesion load [79], suggesting that LDL abnormalities may not only reflect but also exacerbate inflammatory and neurodegenerative processes.

Findings for triglycerides (TG) remain inconsistent, with some studies reporting no significant differences from controls, while others observe elevated TG correlating with worse disability [3,17,21,22]. Non-HDL cholesterol—representing all apoB-containing lipoproteins—has more consistently been found elevated in MS and correlates with disability measures [17,80]. Increased VLDL concentrations in some MS cohorts may contribute to LDL elevations through metabolic conversion and further link peripheral lipid processing with central inflammatory activity [22,79].

At present, CSF biomarkers used in the clinical diagnosis and monitoring of multiple sclerosis are primarily immunological, including oligoclonal IgG bands and kappa free light chains [2]. In contrast, lipid-related markers in CSF—such as oxysterols, including 24S-OHC, or comprehensive lipidomic profiles—remain largely confined to the research setting [38,50,63,81]. Although these lipid markers provide valuable mechanistic insight into CNS cholesterol turnover and neurodegeneration, they are not yet implemented in routine clinical practice.

**Figure 2:**
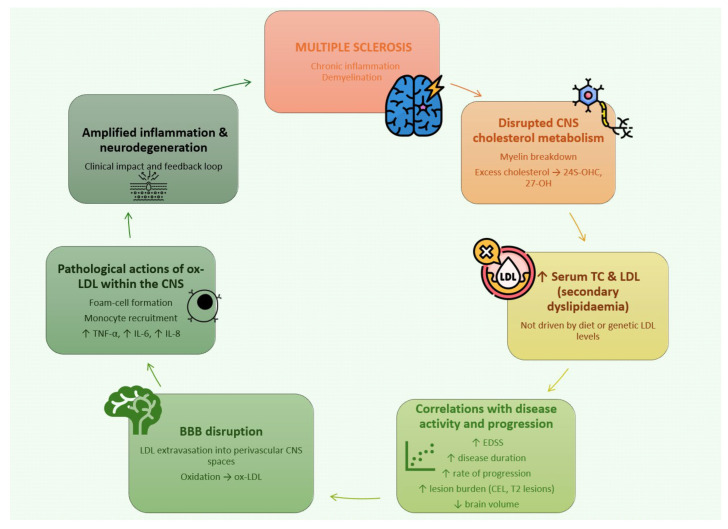
Schematic overview of the mechanistic cascade in multiple sclerosis, depicting disrupted CNS cholesterol metabolism, secondary dyslipidaemia, ox-LDL–driven inflammatory responses, and their contribution to blood–brain barrier injury and clinical worsening. ↑—increased, ↓—decreased.

Figure 2 summarizes the impact of lipid alteration in MS.

### 4.2. HDL in MS

HDL has increasingly been recognized as a key modifier of inflammatory activity, BBB stability, and neurodegeneration in MS. Although serum HDL levels vary across studies, converging biochemical, electrophysiological, and neuroimaging evidence suggests that both HDL concentration and HDL functionality are relevant to disease activity and clinical outcomes.

Early clinical studies reported elevated HDL levels in RRMS compared with healthy controls, along with associations between higher HDL and reduced inflammatory MRI activity, including fewer and smaller contrast-enhancing lesions [3,17,60,75]. However, other cohorts—particularly those including older patients or individuals with SPMS/PPMS—demonstrated reduced HDL and apoA-I, suggesting that HDL may decline with disease progression or chronic inflammation [21,82,83]. A recent meta-analysis encompassing 1692 participants showed that RRMS patients overall have significantly lower HDL-C than healthy controls, with higher TC and TG and no difference in LDL-C, supporting a model in which dysregulated lipid homeostasis accompanies established disease [55].

HDL has been repeatedly linked to radiological markers of neuroprotection. Higher HDL associates with lower lesion burden, reduced risk of future CEL formation, better cerebral perfusion, and slower gray matter atrophy during longitudinal observation [3,56,60,84]. Baseline HDL levels also predict clinical trajectories: RRMS patients with lower HDL exhibit a greater likelihood of conversion to SPMS, underscoring the potential prognostic value of HDL [56].

Mechanistically, HDL contributes to BBB stability, a fundamental determinant of MS pathology. Higher HDL and apoA-I correlate with lower cerebrospinal fluid (CSF) protein, albumin, IgG, and reduced levels of activated immune cells, indicating less BBB leakage [58]. This is consistent with HDL’s ability to suppress endothelial VCAM-1 expression and limit immune cell transmigration, and aligns with findings in Alzheimer’s disease, where high HDL similarly associates with reduced BBB permeability [62,85].

Beyond concentration, HDL functionality plays a central role. Under oxidative stress, HDL becomes dysfunctional, losing its antioxidative and anti-inflammatory capacity. It was demonstrated that HDL is dysfunctional in RRMS, with impaired cholesterol acceptance and reduced anti-inflammatory activity, particularly in individuals with low BMI [22]. The clinical importance of HDL dysfunction is supported by other reaserch: patients with optic neuritis exhibited elevated MPO/PON ratios, strongly correlating with delayed PRVEP P100 latencies, despite no differences in HDL levels [59]. This indicates that dysfunctional HDL may impair remyelination and axonal conduction.

HDL’s involvement in cholesterol trafficking further links it to MS pathophysiology. During demyelination, cholesterol efflux from glial cells increases and is mediated through LXR-dependent pathways that upregulate apoA-I, ABCA1, and ABCG1 to facilitate reverse cholesterol transport. This compensatory HDL response may initially mitigate inflammatory injury, but persistent oxidative and inflammatory stress can eventually impair HDL structure and function [40]. Reduced HDL in progressive forms of MS may therefore reflect exhaustion of these protective mechanisms.

Disease-modifying therapies can alter HDL levels. Fingolimod and dimethyl fumarate increase HDL and apoA-I, correlating with reduced relapse rates and slower disability accumulation [57,86]. Conversely, dysfunctional HDL markers highlight the need for therapeutic strategies aimed not only at raising HDL quantity but also restoring HDL quality.

**Figure 3:**
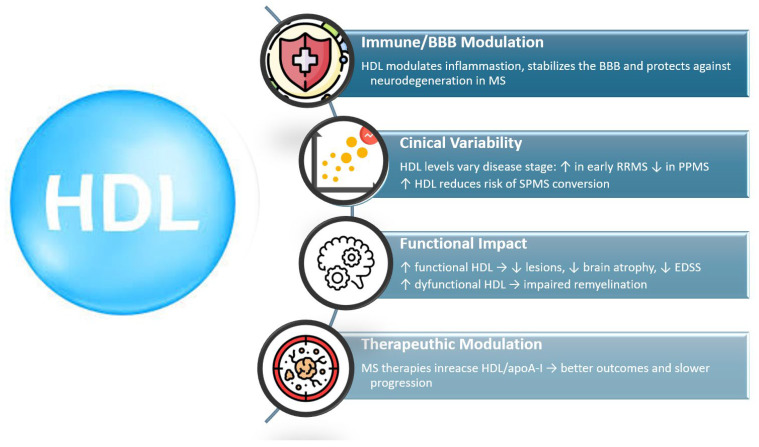
HDL as a key modifier of multiple sclerosis pathology, showing its roles in inflammation control, BBB protection, neuroprotection, cholesterol trafficking, dysfunction under oxidative stress, and therapeutic enhancement. ↑—increased, ↓—decreased.

Figure 3 summarizes the role of HDL in MS.

### 4.3. Lipid Dysregulation and Cognitive Impairment in MS

Cognitive impairment (CI) affects a substantial proportion of individuals with MS and reflects underlying neurodegeneration, white-matter disruption, and failure of compensatory mechanisms. Emerging evidence demonstrates that lipid abnormalities—both classical serum lipids and broader cholesterol-pathway biomarkers—are closely associated with cognitive outcomes in MS.

Several studies indicate that elevated LDL-C and an unfavorable LDL/HDL balance are linked to poorer cognitive performance. In a large cross-sectional study, it was found that Symbol Digit Modalities Test (SDMT) and Nine-Hole Peg Test (NHPT) results correlated with multiple cholesterol-pathway biomarkers (CPB), including LDL-C, ApoB, the TC/HDL-C ratio, and the ApoB/ApoA-I ratio. A higher proportion of LDL relative to HDL was consistently associated with worse processing speed and manual dexterity, and these associations persisted after adjustment for brain parenchymal volume, suggesting that the effects of CPB extend beyond global atrophy [19].

Systematic evidence also supports a role for cholesterol dysregulation in CI. A meta-analysis demonstrated that total cholesterol is negatively associated with global cognition assessed by the Montreal Cognitive Assessment (MoCA), and that 24S-OHC—a marker of CNS cholesterol turnover—is associated with SDMT performance [87]. These findings implicate impaired cholesterol handling and neurodegeneration in cognitive decline.

Neuroimaging studies further reinforce this link. Studies show that higher HDL-C paradoxically associates with increased brain atrophy and T2 lesion burden, while LDL-C demonstrates weaker but directionally similar relationships [76]. Such structural changes are established determinants of CI in MS.

**Figure 4:**
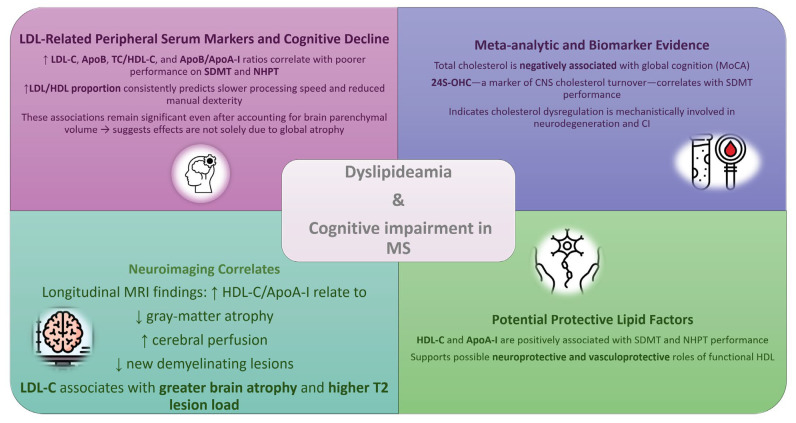
LDL-related markers link to poorer cognitive and motor performance, while cholesterol-turnover biomarkers and MRI measures indicate structural correlates of cognitive decline. Functional HDL (HDL-C, ApoA-I) may exert protective effects. Abbreviations: LDL-C—low-density lipoprotein cholesterol; HDL-C—high-density lipoprotein cholesterol; ApoA-I/ApoB—apolipoproteins A-I and B; TC—total cholesterol; 24S-OHC—24-S-hydroxycholesterol; SDMT—Symbol Digit Modalities Test; NHPT—Nine-Hole Peg Test; CI—cognitive impairment,↑—increased, ↓—decreased.

Conversely, HDL-C and ApoA-I have shown protective associations with SDMT and NHPT. These findings align with prior work linking HDL to preserved BBB integrity and reduced neurodegeneration. Longitudinal MRI studies demonstrate that higher HDL-C or ApoA-I levels correlate with slower gray-matter atrophy, improved cerebral perfusion, and fewer demyelinating lesions, suggesting a potential neuroprotective or vasculoprotective role [56,60,84]. Figure 4 summarizes the impact of lipid alterations on cognitive function in MS patients.

### 4.4. Impact of Lifestyle on Lipid Alteration in MS

Growing evidence indicates that lifestyle behaviors—including dietary patterns, physical activity, and metabolic health—interact with lipid homeostasis and may influence disease expression in MS. Although dyslipidaemia in MS appears to arise primarily from CNS-driven mechanisms associated with demyelination and altered cholesterol turnover rather than dietary intake alone, modifiable lifestyle factors can outline peripheral lipid metabolism and may secondarily modulate disease burden.

Large cohort studies have shown no association between habitual dietary fat intake and MS risk, suggesting that dyslipidaemia in MS is unlikely to be diet-induced in the traditional cardiovascular sense [69]. Nonetheless, targeted dietary interventions may affect lipid fractions relevant to MS pathophysiology. In a randomized 18-month trial, both fasting and ketogenic diets improved cardiometabolic markers—including reductions in adiposity, blood pressure, and lipid indices—such as reductions in LDL-C and TG—across all groups, with exploratory benefits for neurofilament light chain and cognitive performance [88]. These effects may relate to the anti-inflammatory and metabolic consequences of ketosis, improved insulin sensitivity, and modulation of adipokines such as leptin and adiponectin, both implicated in immune dysregulation and disability progression in MS [88].

Multimodal lifestyle interventions integrating a modified Paleolithic diet, structured exercise, neuromuscular electrical stimulation, and stress reduction have similarly demonstrated favorable trends in apolipoprotein profiles (notably reductions in ApoB) and glucose–insulin dynamics, without adverse effects on cardiometabolic status [89]. Improvements in fatigue were associated with changes in insulin resistance, linking metabolic health to patient-reported outcomes.

Exercise—particularly high-intensity interval training (HIIT)—has shown additional benefits for lipid regulation. An 8-week HIIT program significantly reduced total cholesterol and LDL levels while improving lower-limb function and bone metabolism in people with MS [90]. HIIT is thought to enhance skeletal muscle lipid utilization, increase mitochondrial efficiency, and improve insulin sensitivity, thereby contributing to favorable shifts in lipid profiles. Similar improvements have been observed in other metabolic conditions and may help counteract the pro-atherogenic lipid patterns associated with worse MRI and clinical outcomes in MS.

**Figure 5:**
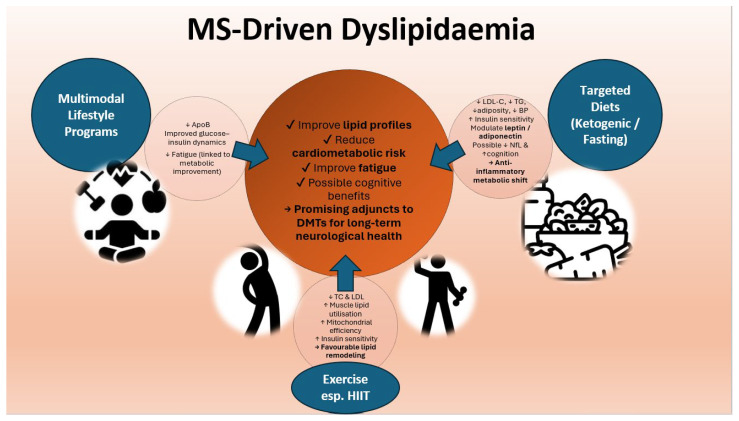
Iconographic summary illustrating the influence of targeted diets, exercise, and multimodal lifestyle interventions on lipid regulation and metabolic–neurological health in multiple sclerosis. Abbreviations: LDL-C, low-density lipoprotein cholesterol; TG, triglycerides; TC, total cholesterol; NfL, neurofilament light chain; HIIT, high-intensity interval training. ↑—increased, ↓—decreased.

Overall, data suggest that diet and physical activity meaningfully influence lipid metabolism in MS, with downstream effects on fatigue, cardiometabolic risk, and possibly cognitive outcomes. While current evidence remains heterogeneous and often limited by small sample sizes, lifestyle interventions represent promising adjunctive strategies that may complement disease-modifying therapies and support long-term neurological health. Figure 5 summarizes impact of lifestyle on lipid alteration in MS.

### 4.5. Statins in MS

Evidence from genetic, mechanistic, and clinical studies indicates that potential benefits of statins in MS arise primarily from cholesterol-independent immunomodulatory mechanisms, rather than from lipid lowering. Mendelian randomization analyses show no causal role for LDL-C or cholesterol biosynthesis pathways in MS risk, and genetically mimicked cholesterol-lowering effects of statins do not reduce susceptibility or severity of MS [20]. In contrast, increased expression of RAC2 (Ras-related C3 botulinum toxin substrate 2), a Rho GTPase that regulates T-cell activation, dendritic cell migration, and peripheral immune tolerance, is causally associated with lower MS risk, supporting the hypothesis that statins may exert beneficial effects through RAC2-mediated pathways [20].

Clinical trial data reflect this dissociation. Meta-analyses of randomized controlled trials using standard-dose statins, typically used for hypercholesterolaemia, have not shown meaningful benefit on relapse rate, disability progression, or MRI outcomes in MS [91,92,93,94]. However, trials employing high-dose simvastatin—particularly in progressive MS—have demonstrated reduced brain atrophy and slowed clinical worsening [95,96,97]. Notably, reductions in total cholesterol among participants with elevated baseline levels correlated with improved neurological outcomes, suggesting a possible metabolically defined responder subgroup.

Genetic evidence that lifelong elevated HDL-C increases MS risk further underscores the complexity of lipid–immune interactions and challenges the assumption that raising HDL-C is universally beneficial [20]. Most statin trials have not included detailed lipidomics or assessments of HDL functionality, limiting mechanistic interpretation.

Overall, current evidence indicates that statins do not modify MS through classical lipid-lowering pathways, but may exert beneficial effects via Rho GTPase–linked immune modulation, particularly RAC2. Ongoing studies, including MS-STAT2, are expected to clarify the therapeutic relevance of these pathways and identify whether specific metabolic or immunological profiles predict treatment response.

In contrast to statins, other cholesterol-lowering agents have not been systematically evaluated in randomized clinical trials in MS. Genetic evidence indicates that dyslipidaemia observed in MS is not causally related to classical atherosclerotic mechanisms but rather reflects secondary consequences of chronic neuroinflammation and disrupted CNS cholesterol metabolism [20]. Nevertheless, selected lipid-modifying agents that primarily target immunometabolic and lipid-signaling pathways rather than cholesterol reduction per se have attracted interest. In particular, agonists of peroxisome proliferator-activated receptor-α (PPAR-α), such as fenofibrate, have demonstrated anti-inflammatory, antioxidant, and neuroprotective effects in preclinical models of MS and related neuroinflammatory conditions. Importantly, the proposed benefits of PPAR-α activation appear to be mediated by modulation of immune responses and cellular metabolism rather than by lowering circulating cholesterol levels, underscoring the relevance of lipid signaling pathways as potential therapeutic targets in MS [98].

Beyond classical cholesterol-lowering approaches, increasing attention has been directed toward therapeutic strategies targeting lipid signaling pathways. Preclinical studies demonstrate that modulation of endocannabinoid-like lipid mediators, including palmitoylethanolamide, or inhibition of their metabolic enzymes, such as N-acylethanolamine acid amidase, can attenuate neuroinflammation and disease severity in experimental models of MS [65,67,99]. Importantly, clinical evidence indicates that treatment with dimethyl fumarate is associated with dynamic modulation of endocannabinoid levels in patients with MS, suggesting that lipid signaling pathways may already be indirectly targeted by approved disease-modifying therapies [54]. Although these approaches have not yet been translated into clinical trials in MS, they highlight lipid signaling—not cholesterol reduction per se—as a promising direction for future therapeutic development.

## 5. Materials and Methods

This narrative review draws upon a selection of research studies examining lipid profile alterations in multiple sclerosis (MS) and their potential relevance to disease mechanisms and progression. Relevant original research articles, editorials, and reviews published up to November 2025 were identified through the PubMed database. The literature search utilized keywords including “lipids”, “cholesterol”, “HDL”, “LDL”, “apolipoproteins”, “lipoproteins”, “multiple sclerosis”, “neuroinflammation”, and “cholesterol metabolism” to capture studies addressing the interactions between systemic lipid abnormalities, demyelination, and MS pathogenesis.

## 6. Conclusions

Lipid dysregulation has emerged as a significant but complex contributor to the pathobiology of multiple sclerosis. Although dyslipidaemia in MS does not appear to originate from traditional cardiovascular mechanisms or dietary fat intake, converging evidence demonstrates that disturbances in cholesterol metabolism, lipoprotein particle composition, and lipid-mediated immune signaling are closely intertwined with inflammatory activity, neurodegeneration, and clinical progression. Altered CNS cholesterol turnover—reflected by changes in 24S-hydroxycholesterol—together with peripheral abnormalities in LDL, HDL, apoA-I, and apoB, suggests that myelin injury and impaired lipid handling form a bidirectional interface between central and systemic compartments.

Elevated LDL-C and higher LDL/HDL ratios correlate with worse disability, greater lesion burden, and poorer cognitive performance, implicating pro-atherogenic lipoproteins in amplifying inflammatory cascades and axonal injury. In contrast, HDL quantity and function appear variably protective: while higher HDL and apoA-I associate with reduced BBB permeability and slower atrophy, dysfunctional HDL—particularly under oxidative stress—may impair remyelination and conduction.

Lifestyle and metabolic factors can modify lipid profiles and may offer complementary therapeutic relevance. Dietary interventions, ketogenic or fasting regimens, and structured physical activity—especially high-intensity interval training—demonstrate favorable shifts in atherogenic lipids, adipokines, and insulin sensitivity, alongside potential benefits for fatigue, mood, and cognition.

Finally, evidence indicates that statins exert any potential benefit in MS through cholesterol-independent mechanisms, notably RAC2-related immunomodulation, rather than LDL reduction. High-dose simvastatin trials in progressive MS warrant further evaluation.

Together, these findings underscore lipid metabolism as an important mechanistic and potentially modifiable domain within MS biology, meriting integration into future diagnostic, prognostic, and therapeutic strategies.

## 7. Highlights

1. Lipid abnormalities in multiple sclerosis are secondary to demyelinating processes rather than an independent comorbid condition.
2. Elevated LDL and total cholesterol levels correlate with greater disability and faster disease progression.
3. HDL exerts neuroprotective effects.
4. Improved physical activity and dietary habits enhance lipid profiles and reduce disability severity.

## Figures and Tables

**Table 1:** Alterations in major lipid classes in multiple sclerosis.

Lipid Class	Main Lipid Species/Markers	Direction of Change in MS	Biological Relevance in MS	Key References
Total cholesterol & LDL-related lipids	Total cholesterol (TC), LDL-C, apoB, apoB/apoA-I ratio	↑ (especially with disease duration, disability, and progression)	Secondary dyslipidaemia associated with demyelination and disturbed CNS cholesterol turnover; correlated with EDSS, disease duration, and cognitive and motor impairment	[3,15,16,18,19,20,21,55,56]
HDL-related lipids	HDL-C, apoA-I, HDL particle number/function	↓ or functionally impaired (stage- and phenotype-dependent)	HDL quantity and functionality influence inflammation, BBB integrity, cholesterol efflux, and neuroprotection; dysfunctional HDL reported in MS	[3,16,17,19,22,57,58,59,60,61,62]
Oxysterols	24S-hydroxycholesterol (24S-OHC), 27-hydroxycholesterol	↑ during acute demyelination and relapses; ↓ in chronic neurodegeneration	Reflect CNS cholesterol turnover and myelin injury; potential markers of disease activity and neurodegeneration	[16,32,39,63]
Cholesterol esters	Cholesteryl esters (CE)	↑ (mainly experimental evidence)	Excess myelin-derived cholesterol is esterified in glial cells during demyelination, indicating altered intracellular lipid handling and inflammatory responses	[25,34]
Phospholipids	Phosphatidylcholine, phosphatidylethanolamine, phosphatidylserine	Altered composition	Reflect membrane remodeling, myelin breakdown, and neurodegeneration	[4,49,50,51,52,64]
Endocannabinoids & related lipids	Palmitoylethanolamide (PEA), N-acylethanolamines	Dysregulated	Lipid signaling mediators modulating neuroinflammation, oxidative stress, and BBB stability	[54,65,66,67]
Triglycerides & TG-rich lipoproteins	Triglycerides, VLDL, TG-rich particles	↑ (subset of patients)	Reflect metabolic dysregulation and may contribute to vascular and inflammatory burden in MS	[3,16,17,21,22,55]

Reported lipid alterations in multiple sclerosis vary across studies, disease stages, and clinical phenotypes. Available evidence supports the concept that serum lipid abnormalities in MS are predominantly secondary to demyelination, chronic neuroinflammation, and altered CNS cholesterol turnover rather than primary metabolic dyslipidaemia. ↑—increased serum level, ↓—decreased serum level.

## Data Availability

No new data were created or analyzed in this study. Data sharing is not applicable to this article.
